# Whole genome sequencing of phage resistant *Bacillus anthracis* mutants reveals an essential role for cell surface anchoring protein CsaB in phage AP50c adsorption

**DOI:** 10.1186/1743-422X-9-246

**Published:** 2012-10-26

**Authors:** Kimberly A Bishop-Lilly, Roger D Plaut, Peter E Chen, Arya Akmal, Kristin M Willner, Amy Butani, Shakia Dorsey, Vishwesh Mokashi, Alfred J Mateczun, Carol Chapman, Matroner George, Truong Luu, Timothy D Read, Richard Calendar, Scott Stibitz, Shanmuga Sozhamannan

**Affiliations:** 1 NMRC-Frederick, 8400 Research Plaza, Ft. Detrick, MD 21702, USA; 2 Division of Bacterial, Parasitic, and Allergenic Products, Center for Biologics Evaluation and Research, Food and Drug Administration, 8800 Rockville Pike, Bethesda, MD 20892, USA; 3 Department of Human Genetics, Department of Medicine, Division of Infectious Diseases, Emory University School of Medicine, Atlanta, GA 30322, USA; 4 Department of Molecular and Cell Biology, University of California Berkeley, Berkeley, CA 94720, USA

**Keywords:** Phage resistance, WGS, Mutation mapping, SNP, *B. anthracis*

## Abstract

**Background:**

Spontaneous *Bacillus anthracis* mutants resistant to infection by phage AP50c (AP50^R^) exhibit a mucoid colony phenotype and secrete an extracellular matrix.

**Methods:**

Here we utilized a Roche/454-based whole genome sequencing approach to identify mutations that are candidates for conferring AP50c phage resistance, followed by genetic deletion and complementation studies to validate the whole genome sequence data and demonstrate that the implicated gene is necessary for AP50c phage infection.

**Results:**

Using whole genome sequence data, we mapped the relevant mutations in six AP50^R^ strains to *csaB*. Eleven additional spontaneous mutants, isolated in two different genetic backgrounds, were screened by PCR followed by Sanger sequencing of the *csaB* gene. In each spontaneous mutant, we found either a non-synonymous substitution, a nonsense mutation, or a frame-shift mutation caused by single nucleotide polymorphisms or a 5 base pair insertion in *csaB*. All together, 5 and 12 of the 17 spontaneous mutations are predicted to yield altered full length and truncated CsaB proteins respectively. As expected from these results, a targeted deletion or frame-shift mutations introduced into *csaB* in a different genetic background, in a strain not exposed to AP50c, resulted in a phage resistant phenotype. Also, substitution of a highly conserved histidine residue with an alanine residue (H270A) in CsaB resulted in phage resistance, suggesting that a functional CsaB is necessary for phage sensitivity. Conversely, introduction of the wild type allele of *csaB in cis* into the *csaB* deletion mutant by homologous recombination or supplying the wild type CsaB protein *in trans* from a plasmid restored phage sensitivity. The *csaB* mutants accumulated cell wall material and appeared to have a defective S-layer, whereas these phenotypes were reverted in the complemented strains.

**Conclusions:**

Taken together, these data suggest an essential role for *csaB* in AP50c phage infection, most likely in phage adsorption. (The whole genome sequences generated from this study have been submitted to GenBank under SRA project ID: SRA023659.1 and sample IDs: AP50 R1: SRS113675.1, AP50 R2: SRS113676.1, AP50 R3: SRS113728.1, AP50 R4: SRS113733.1, AP50 R6: SRS113734.1, JB220 Parent: SRS150209.1, JB220 Mutant: SRS150211.1).

## Background

The evolutionary arms race between bacteria and their phages, giving rise to resistance and counter resistance mechanisms, shapes the bacterial and phage population size and composition as well as the evolutionary success of these two entities [1,2]. The major phage resistance mechanisms exhibited by bacteria and the strategies adopted by phages to counter these resistance mechanisms have been extensively treated in two recent reviews [1,2]. Understanding phage resistance mechanisms is critical in developing phage therapy applications.

*Bacillus anthracis,* a category A biothreat agent, is a spore-forming Gram-positive bacterium of the *Bacillus cereus sensu lato* group. It is a zoonotic soil bacterium that infects animals and occasionally humans, causing the disease anthrax. The October-November 2001 bioterrorism attacks using mail laced with anthrax spores brought about renewed interest in research on *B. anthracis*. The prospect of biothreats using *B. anthracis* and the possibility of naturally emergent or deliberately created antibiotic resistant *B. anthracis* call for highly integrated and enhanced technological platforms for diagnosis and detection of this organism. This need is best illustrated in the case of a bacterial bioterror attack, where timely detection and intervention with countermeasures such as antibiotic therapy are paramount in preventing fatal consequences. Bacteriophages have been and still remain useful tools for bacterial species and strain differentiation [3-6], although evidence of successful application of phage therapy is still sparse in Western medicine [7].

Recently, the inherent binding specificity and lytic action of bacteriophage-encoded enzymes called lysins have been exploited for the rapid detection and killing of *B. anthracis*[8]. These authors demonstrated that the PlyG lysin isolated from the γ phage of *B. anthracis* specifically kills *B. anthracis* isolates and other members of the *B. anthracis* “cluster” of bacilli *in vitro* and *in vivo*. Both vegetative cells and germinating spores were shown to be susceptible. The lytic specificity of PlyG was also exploited as part of a rapid method for the identification of *B. anthracis*[8]*.* Thus, PlyG is a useful tool not only for the treatment but also for the detection of *B. anthracis*.

The standard diagnostic tests for suspected *B. anthracis* as recommended by the Centers for Disease Control and Prevention (CDC) are as follows: presumptive identification to genus level (*Bacillus* family of organisms) requires Gram stain and colony identification. Presumptive identification to species level (*B. anthracis*) requires tests for motility, lysis by γ phage, capsule production and visualization, hemolysis, wet mount and malachite green staining for spores. Confirmatory identification of *B. anthracis* may include lysis by γ phage, capsular staining, and direct fluorescent antibody (DFA) testing on capsule antigen and cell wall polysaccharide. Thus, testing for γ phage sensitivity is an integral part of *B. anthracis* identification [9]. γ phage exhibits a fairly narrow host range but several *B. cereus* strains (e.g., ATCC 4342) have been shown to be sensitive to infection by this phage [8,10-12]. Several phages (CP51, CP54 and TP21) isolated from *B. cereus* and *B. thuringiensis* strains have been successfully used for transducing chromosomal markers and plasmids between *B. anthracis* strains [13-17]. However, their utility as *B. anthracis* diagnostic phages is limited because of their broad host range.

Bacteriophage AP50 was isolated from soil using *B. anthracis* Sterne as the host [18]. Originally it was thought to be an RNA phage but was later shown to contain double stranded DNA and phospholipid [19]. AP50 was also shown to have a narrow host range; only one third of the 34 *B. anthracis* strains and none of the 52 strains belonging to 6 different *Bacillus* species were susceptible to infection by AP50 [20]. Nine major structural proteins were identified on SDS-PAGE gels. The molecular weight of the phage DNA was estimated to be 9 × 10^6^ daltons [21]. Treatment with organic solvents such as chloroform (5%) and ether (25%) for 30 minutes inactivated the phage to a survival of about 1 × 10^-4^[22].

In a recent study, we described the genetic characterization of AP50c, which is a derivative of AP50 [23]. In contrast to the original report [20] on AP50, AP50c exhibited a much narrower host range, infecting 111 of the 115 *B. anthracis* strains (97%) and none of the 100 *B. cereus sensu lato* strains. We also determined the genome sequence by 454 pyrosequencing and compared it to other *Tectiviridae* phages [23]. The genome size was determined to be 14,398 bp in length and shown to be highly similar to other *Tectiviridae* phages infecting Gram-positive bacteria in genetic organization and encoded proteins. We identified two mutations in the AP50 genome that were responsible for the control of lysogenic/lytic lifestyles and consequently the phenotypic shift from turbid to clear plaque morphology. Based on these properties, we proposed that AP50c could be used as an additional diagnostic and therapeutic tool for *B. anthracis*. Before embarking on phage therapy using AP50c, we sought to characterize the phage resistance mechanisms in *B. anthracis* in response to AP50c infection. In order to address this problem, we took an approach that began with whole genome scanning and concluded with genetic validation. Initially, we isolated a number of *B. anthracis* mutants that were spontaneously resistant to killing by AP50c and observed that these mutants produced an extracellular material that coats the cell surface [23]. We hypothesized that this may be a bacterial adaptive resistance mechanism masking the bacterial receptor of AP50c.

In the current study, we have mapped multiple bacterial mutations responsible for spontaneous AP50c resistance. Historically, mapping mutations responsible for specific phenotypes has involved transfer of the mutation with a closely linked selectable marker via one of the three conventional methods: transduction, conjugation or transformation. Physical mapping has involved cloning and sequencing of the genetically mapped gene using traditional cloning vectors and methods. The recent development of rapid, cost-effective sequencing of bacterial genomes using second-generation sequencers or array-based comparative genome resequencing has accelerated the mapping of causal variations in bacterial as well as in other model system genomes [24-26]. We recently demonstrated the use of 454- generated draft sequences to map mutations conferring various phenotypes, and in that study a putative mutation associated with AP50c resistance was mapped to *csaB*[27], although the link between genotype and phenotype was not verified in that report. CsaB (for cell surface anchoring) protein is involved in pyruvylation of the peptidoglycan-associated polysaccharide, and this modification is necessary for binding of the S-layer homology (SLH) domain. Thus, CsaB is critical for the non-covalent anchoring of SLH-domain containing proteins onto the cell surface [28]. In the current study, we explore the relevance of the preliminary finding of a potential linkage between the *csaB* gene and AP50c phage resistance by isolating and characterizing additional spontaneous mutants and, by using a recently described method [29], validating the WGS data by recreating *csaB* point and deletion mutations in a different genetic background and showing phage AP50c resistance. Furthermore, we restored sensitivity to AP50c infection in the mutants by complementing the defect in the Δ*csaB* mutant *in cis* as well as *in trans,* thereby establishing an essential role of *csaB* in phage infectivity.

## Results and discussion

### Whole genome pyrosequencing and identification of putative variants in AP50^R^ mutants

**Figure 1:**
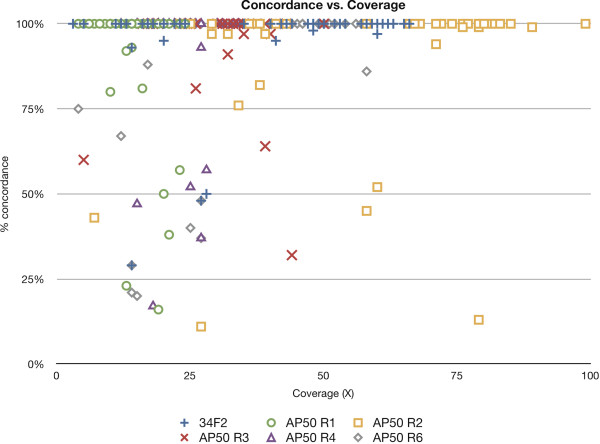
**Filtration of true positive variations from false positive variations by percent concordance and coverage.** The graph displays 260 high quality (HQ) variations in the genome sequences (34F_2_ and its derivatives) produced by GS Reference Mapper software as a function of percentage concordance. The graph indicates all the high-quality variations in the six genomic sequences obtained in this work. The variations from the six strains are indicated by different symbols. AP50 R1, AP50 R2, AP50 R3, AP50 R4 and AP50 R6 are S-R1, S-R2, S-R3, S-R4 and S-R6 respectively.

We took a whole genome sequencing (WGS) approach to map the mutations and identify the gene(s) responsible for the phage resistant phenotype of the AP50^R^ mutants. We have shown previously that the draft sequences generated by Roche/454 shotgun pyrosequencing can be used efficiently to map mutations responsible for various phenotypes [27]. We generated whole genome sequence data of five spontaneous AP50^R^ mutant strains and compared them to that of the AP50c sensitive parent strain. We reasoned that the causal variation of phage resistance might occur in the same gene (for example in the gene encoding the bacterial receptor of the phage) in all five mutants (S-R1, S-R2, S-R3, S-R4, and S-R6), assuming that there is a single pathway to resistance. Hence, we searched for a common variation in all five mutants that is not present in the phage-sensitive parent strain. The phage-sensitive parent strain (Sterne 34F_2_) and the five mutants were sequenced by FLX or Titanium protocol. Some of the basic statistics of the genomic sequences are presented in Table 1. The estimated average coverage of the genomes ranged from 12X to 46X, and the number of high-quality variations (see Methods) ranged from 46–52 (Table 1). The number of contigs was generally proportional to the Q39 scores; i.e., the lower the Q39 score the lesser the number of contigs. Among the high-confidence variations, the most likely true positive variations (high-quality) were selected for by applying the following criterion: variations that had a percentage concordance of more than 75% variant base in the reads spanning any given position. Figure 1 shows the variations as a function of the percentage concordance of the variant reads. There were a total of 242 high-quality variations across all the five AP50^R^ mutants, and of these 215 showed 75% or more concordance. Among these, 200 were identity-by-descent variations. The genomic locations of the 15 unique variations along with their annotations are shown in Table 2. Among all the genes encoding variations, only two were mutated in all five resistant strains and not in the sensitive strain. These were BAS0840 (*csaB*), which encodes a cell-surface anchoring protein [28] and BAS3946, which encodes a 2, 3-diketo-5-methylthiopentyl-1-phosphate enolase.

**Table 1:** **Statistics of genome sequence of wild type and mutant *B. anthracis *strains**

**Strain**	**Runs**	**Run type**	**Length of assembled genome (bp)**	**% ≤ Q39**	**Mean read length (bp)**	**Number of contigs**	**Depth of Coverage**	Number of putative HQ* variants
34F_2_	1	FLX	5,356,131	0.06	274	101	23	48
S-R1	1	FLX	5,346,471	0.37	245	227	12	47
S-R2	2	FLX	5,355,072	0.02	274	60	46	50
S-R3	1	FLX	5,355,679	0.04	283	92	28	46
SR-4	1	FLX	5,347,433	0.06	282	102	21	47
SR-6	1	FLX	5,349,716	0.05	277	98	26	52
JB220	1	Titanium	5,349,773	0.39	264	111	23	43
J-R1	1	Titanium	5,348,852	0.59	269	128	22	45

*HQ, high confidence and high concordance (≥75%).

**Table 2:** **Location of true positive variations in genomes of mutant*B. anthracis*compared to reference genome**

**Strain**	**Start position**	**Stop position**	**Reference allele**	**Sample allele**	**Locus description**	**Coverage**	**Percent Concordance**
S-R1	894,910	894,910	-	G	CsaB protein	10	100
	3,894,626	3,894,626	G	A	2,3-diketo-5-methylthiopentyl-1-phosphate enolase	8	100
S-R2	210,679	210,679	T	G	intergenic	29	97
	895,097	895,097	-	G	CsaB protein	38	82
	2,139,026	2,139,026	G	T	sensor histidine kinase	34	76
	3,894,626	3,894,626	G	A	2,3-diketo-5-methylthiopentyl-1-phosphate enolase	24	100
S-R3	895,097	895,097	-	G	CsaB protein	23	100
	3,894,626	3,894,626	G	A	2,3-diketo-5-methylthiopentyl-1-phosphate enolase	19	100
S-R4	895,066	895,066	A	-	CsaB protein	18	100
	3,894,626	3,894,626	G	A	2,3-diketo-5-methylthiopentyl-1-phosphate enolase	19	100
S-R6	160,743	160,743	A	-	pX01	58	86
	895,083	895,083	-	GCTTA	CsaB protein	17	88
	1,330,746	1,330,746	G	CGGT	intergenic	4	75
	3,894,626	3,894,626	G	A	2,3-diketo-5-methylthiopentyl-1-phosphate enolase	14	100
	4,303,652	4,303,652	-	AC	solute-binding family 5 protein	20	100
J-R1	68,981	68,981	A	-	hypoxanthine-guanine phosphoribosyltransferase	33	97
	895,436	895,436	G	T	CsaB protein	23	96

### Mutation in *csaB* is responsible for phage AP50c resistance

**Figure 2:**
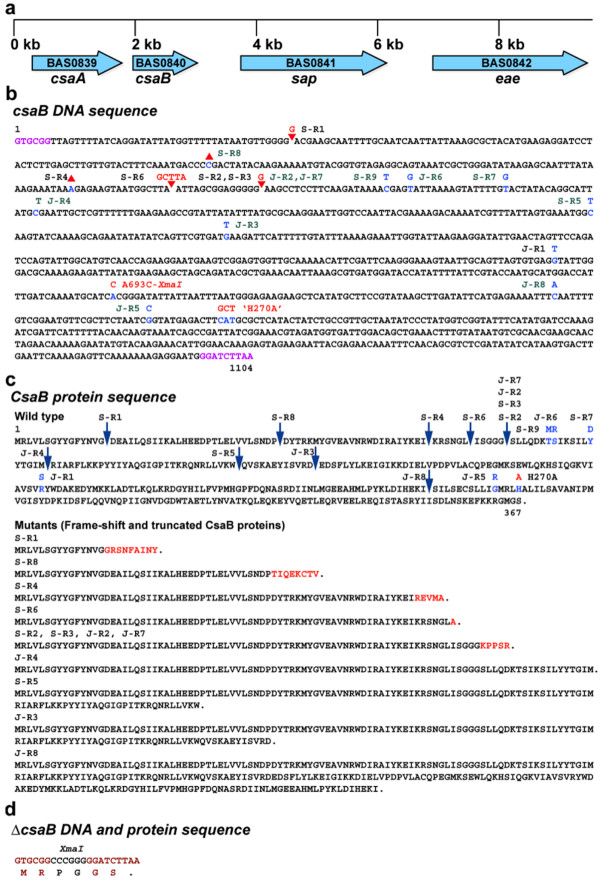
**Genetic map of *csa *operon, DNA and protein sequences of *csaB*.a**) Genetic map of the *csaB* region on *B. anthracis* chromosome. The block arrows indicate the genes and the direction of transcription of the different genes. **b**) Nucleotide sequence of the wild type *csaB* gene and the location and nucleotide changes in the AP50^R^ mutants. Insertions and deletions are indicated by the red arrowheads and inverted arrowheads respectively. The various strains are indicated next to the mutated sites: derivatives of Sterne 34F_2_ and JB220 are indicated by the prefixes S and J respectively. The mutant loci labeled in black were discovered by WGS and those in green by Sanger sequencing of *csaB* PCR fragments. The altered bases are indicated in blue with the changed base above the wild type. A693C and H270A mutations were genetically engineered mutants. The sequences at the beginning and end of the gene marked in pink are the junctions the *csaB* deletion mutant. **c**) Amino acid sequence of wild type and mutant CsaB proteins. The sites of truncation of CsaB protein due to frame-shift and nonsense mutations are indicated by blue downward arrows. The amino acid changes in non-synonymous mutants are indicated in blue above the wild type residue. The amino acid sequences of the frame-shift and truncated proteins are shown. The residues in red are the additional residues added due to the frame-shift before truncation of the mutant proteins. **d**) DNA and protein sequence of the *csaB* gene in *csaB* deletion mutant.

**Figure 3:**
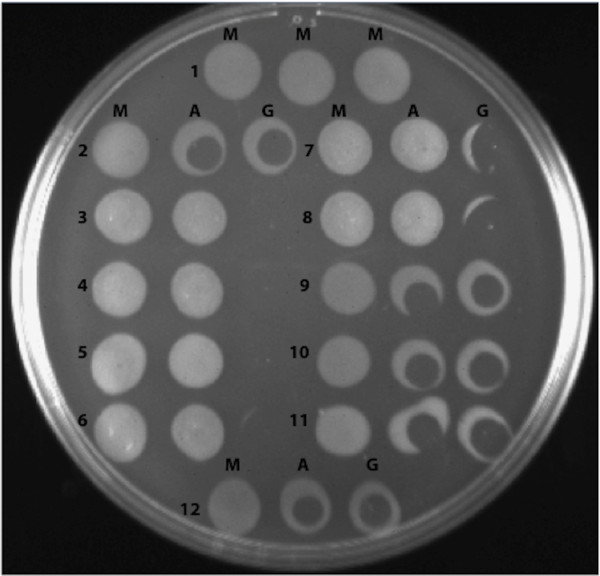
**Phage sensitivity spot test on Sterne 7702 and various derivatives.** For each row the left spot was spotted with medium (M), the middle spot received AP50c (A) and the right spot was spotted with γ (G). The clearing in the bacterial spot indicates infection and killing of bacteria. In rows 3 through 6, the γ spots completely cleared the bacterial spots. The various strains are as follows: 1) and 2) BA663 (wild type); 3) BAP350 (Δ*csaB*); 4) BAP356 (*csaB* ∇G234); 5) BAP366 (*csaB* ∇G46); 6) BAP411 (*csaB* ∇GCTTA219); 7) BAP435 (*csaB* ΔA203); 8) BAP503 (*csaB* H270A); 9) BAP533 (*csaB* A693C); 10) BAP553 (ΔBAS3946); 11) BAP560 (BAS3946 G1024A); 12) BGBN 001 (*ΔcsaB*/pBGBN1003)

Of the two mutated genes present in the AP50^R^ strains identified by WGS, BAS0840 (*csaB*) was determined to be the most probable candidate gene for phage resistance based on the following criteria: 1) In each of the five mutants (S-R1, S-R2, S-R3, S-R4 and S-R6) for which WGS was performed, a frame-shift mutation (∇G46 in S-R1, ΔA203 in S-R4, ∇GCTTA219 in S-R6, ∇G234 in S-R2 and S-R3) (Figure 2) was identified, which in all cases resulted in truncation of CsaB protein and possibly inactivation of its function. 2) Four additional spontaneous resistant mutants from the same genetic background (34F_2_) were screened by *csaB* PCR followed by Sanger sequencing of the *csaB* amplicons and were found to carry mutations in *csaB* as well (C385T, T274G, ΔC129, C254T). Similar to the findings in the first set of mutants, the C385T and ΔC129 mutations result in a truncated CsaB protein, whereas the T274G and C254T mutations result in a change from a noncharged (tyrosine) to a charged amino acid (aspartic acid) and a polar hydrophilic to a nonpolar hydrophobic amino acid, respectively. 3) A spontaneous AP50^R^ mutant generated in a different genetic background (JB220) was subjected to WGS and found not to carry the enolase (BAS3946) mutation, but it did carry a mutation in *csaB* (G573T), which results in a change from a positively charged (arginine) to a noncharged (serine) residue. 4) Seven additional spontaneous phage resistant mutants in the JB220 background were screened by the *csaB* PCR method described above and also found to carry mutations in *csaB* (G421T, C292T, G796C, T258G, C767A, and ∇G234, the latter of which was found in 2 different mutants). Interestingly, two of these mutations result in a noncharged residue being mutated to a charged residue, and the others result in a truncated *csaB*. These results are summarized in Figure 2 and Table 3. 5) Targeted introduction of mutations in *csaB* in a strain (7702) not previously exposed to AP50c resulted in phage resistance (see below and Table 4). 6) Finally, BAS3946 was deemed unlikely to be involved in phage resistance because the mutation causes a non-synonymous change at G1024A leading to G342S in all five mutants (S-R1, S-R2, S-R3, S-R4 and S-R6) and thus does not result in the loss of the protein. PCR amplification and Sanger sequencing of BAS3946 from all the AP50^R^ mutants showed that the mutation is present only in seven out of nine mutants arising from 34F_2_ background and the parent and not in any of the eight mutants of JB220 background. Additionally, a targeted deletion of BAS3946 or the G1024A point mutation in BAS3946 in a different genetic background (strain 7702) in a strain not exposed to AP50c had no effect on AP50c phage sensitivity (Figure 3).

**Table 3:** **Results of Sanger verification of the various spontaneous AP50**^**R**^**mutants**

**Sanger run ID**	**Strain**	**Sanger data**	**Mutation**	**454 data**	**Implication**	**BAS3946**
csaB 1	34F_2_ parent	WT	None	WT	-	G1024A
csaB 2	S-R1	∇ G between positions 46 and 47	Frame-shift	Same	Truncation of CsaB	G1024A
csaB 3	S-R2	∇ G between positions 233 and 234	Frame-shift	Same	Truncation of CsaB	G1024A
csaB 4	S-R3	∇ G between positions 233 and 234	Frame-shift	Same	Truncation of CsaB	G1024A
csaB 5	S-R4	Δ A at position 203	Frame-shift	Same	Truncation of CsaB	G1024A
csaB 6	S-R5	C385T	Nonsense	ND	Truncation of CsaB	G1024A
csaB 7	S-R6	∇ GCTTA between position 219-220	Frame-shift	Same	Truncation of CsaB	G1024A
csaB 8	S-R7	T274G	Y92D	ND	Noncharged to charged (−)	G1024A
csaB 9	S-R8	Δ C at position 129	Frame-shift	ND	Truncation of CsaB	WT
csaB 10	S-R9	C254 T	T85M	ND	Polar hydrophilic to nonpolar hydrophobic	WT
csaB 11	JB220 parent	WT	None	WT	-	WT
csaB 12	J-R1	G at position 573 to T	R191S	Same	Charged (+) to noncharged	WT
csaB 13	J-R2	∇ G between positions 233 and 234	Frame-shift	ND	Truncation of CsaB	WT
csaB 14	J-R3	G421T	Nonsense	ND	Truncation of CsaB	WT
csaB 15	J-R4	C 292T	Nonsense	ND	Truncation of CsaB	WT
csaB 16	J-R5	G796C	G266R	ND	Noncharged to charged (+)	WT
csaB 17	J-R6	T258G	S86R	ND	Noncharged to charged (+)	WT
csaB 18	J-R7	∇ G between positions 233 and 234	Frame-shift	ND	Truncation of CsaB	WT
csaB 19	J-R8	C767A	Nonsense	ND	Truncation of CsaB	WT

∇ and Δ indicate insertion and deletion of base(s) respectively at the indicated position from the start of the gene. ND- Not Done.

WT- Wild type; G1024A indicates a SNP in BAS3946 at position 1024 from the start of gene from G to A.

**Table 4:** Strains and plasmids used in this study

**Strain**	**Description**	**Source/Reference**
*Escherichia coli* SM10	-	[39]
*Bacillus anthracis* 34F_2_	Sterne pXO1^+^pXO2^-^	Hanna lab
*Bacillus anthracis* BA663	7702 Sterne pXO1^+^pXO2^-^	Koehler lab
*Bacillus anthracis* JB220	7702 ΔBAS2245	Calendar lab
*Bacillus anthracis* S-R1 through S-R9	Spontaneous AP50R mutants	This study
*Bacillus anthracis* J-R1 through J-R8	Spontaneous AP50R mutants	This study
*Bacillus anthracis* BAP350	BA663 Δ*csaB*	This study
*Bacillus anthracis* BAP356	BA663 (AP50R-3)-*csaB* ∇G234	This study
*Bacillus anthracis* BAP366	BA663 (AP50R-1)-*csaB* ∇G46	This study
*Bacillus anthracis* BAP411	BA663 (AP50R-6)-*csaB* ∇GCTTA219	This study
*Bacillus anthracis* BAP435	BA663 (AP50R-4)-*csaB* ΔA203	This study
*Bacillus anthracis* BAP503	BA663 *csaB* H270A	This study
*Bacillus anthracis* BAP533	BAP350 *csaB* A693C creating an *XmaI* site	This study
*Bacillus anthracis* BAP553	BA663 ΔBAS3946	This study
*Bacillus anthracis* BAP560	BA663 with BAS 3946 G1024A mutation	This study
*Bacillus anthracis* BGBN 001	BAP350/pBGBN1003	This study
Plasmid pRP1028	Mutant construction	This study
Plasmid pSS4332	Mutant construction	This study
Plasmid pSW4	*E. coli*- *B. anthracis* shuttle vector	[37]
Plasmid pBGBN1001	pSW4 *NotI*	This study
Plasmid pBGBN1002	pSW4 *oriT*	This study
Plasmid pBGBN1003	pSW4 *oriT csaB*	This study
Plasmid pGOv4	pUC19 Amp^R^, Kan^R^	Gene Oracle
Plasmid pNV36	pGOv4 ΔBAS3946	Gene Oracle
Plasmid pNV37	pGOv4 BAS3946 G1024A	Gene Oracle

∇-insertion and Δ-deletion of base (s) at any given position from the start of the gene; e.g., *csaB* ∇G46 indicates insertion of G base after position 46 (between 46 and 47) from the start of the gene, and *csaB* ΔA203 means deletion of A at position 203 from the start of gene.

### Reconstructed *csaB* point mutants and in-frame deletion of *csaB* result in mucoid colony morphology and phage resistance

In order to unequivocally link the phage resistance phenotype to *csaB* and to rule out the involvement of other mutations that may be present in the original AP50^R^ mutants, we reconstructed four of the point mutations in a different genetic background, in Sterne strain 7702, which was not previously exposed to AP50c (see Methods). All four mutants (∇G46, ΔA203, ∇GCTTA219, ∇G234) gave rise to mucoid colony morphology and phage resistant phenotype similar to the original spontaneous mutants, indicating that *csaB* indeed is involved in phage sensitivity. In addition to these mutants, we created a scarless, markerless in-frame deletion of *csaB* in 7702 background, which also showed mucoid and phage resistant phenotypes (Figure 3).

### Functional CsaB is needed for phage sensitivity

**Figure 4:**
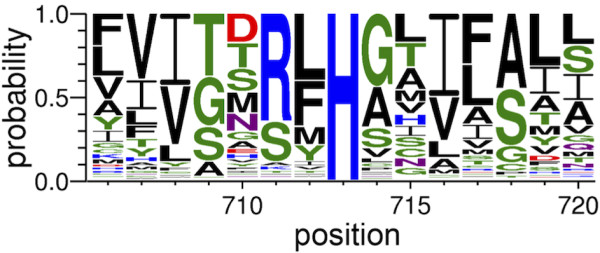
**Amino acid sequence alignment of *csaB *orthologs.** BAS0840 was aligned with the Pfam PF04230 seed alignment, and percent conservation was determined as described in Methods. Only the alignment of the amino acids in the vicinity of histidine 270 of CsaB protein is shown. The full-length alignment is provided in Additional file 1: Figure S1.

Having shown that inactivation of *csaB* results in phage resistance, we wished to rule out the possibility that this result is due to a polar effect of the *csaB* mutations on downstream gene(s) or due to an alteration of regulatory elements in the deletion mutant. *csaA* and *csaB* form an operon upstream of *sap* and *eae* (Figure 2a)*,* which have been shown to be involved in the formation of the cell surface associated S-layer (Sap) or an alternate S-layer (EA1) respectively [28]. In order to test the hypothesis that a functional CsaB is needed for phage sensitivity, we sought to inactivate the protein by mutating a critical amino acid residue while retaining the full-length protein and all the potential upstream and downstream control elements. A protein sequence alignment of 163 known orthologs revealed several conserved residues within CsaB protein (Figure 4 and Additional file 1: Figure S1) of which the histidine at position 270 is 100% conserved. We mutated histidine 270 to an alanine, and the resulting mutant was found to be mucoid and phage resistant (Figure 3), suggesting that a functional CsaB protein is necessary for phage infectivity, and inactivation of the CsaB protein either by truncation or by mutating a critical amino acid residue gives rise to phage resistance.

### Complementation of *csaB* mutants with wild type copy of *csaB*

**Figure 5:**
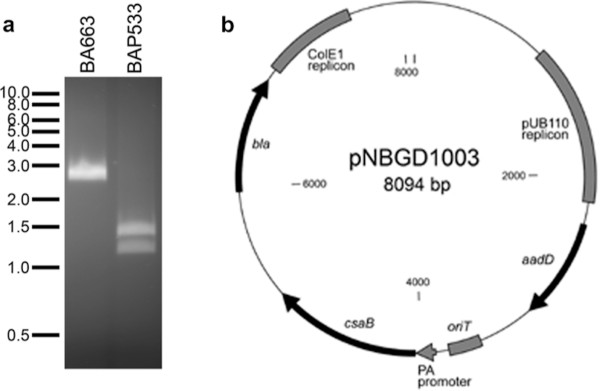
**Complementation of *csaB *mutation.a**) PCR verification of *Xma*I mutant *csaB*. The wild-type sequence of *csaB* was restored in the Δ*csaB* strain with the addition of a silent mutation creating an *Xma*I site (see Methods). The region was amplified by PCR, and PCR products were purified and digested with *Xma*I. The lane labeled BA663 indicates the *csaB* PCR product from wild type strain (7702) after digestion with *Xma*I, whereas the lane labeled BAP533 is the PCR product from the mutant showing the two bands after digestion with *Xma*I. **b**) Genetic map of *csaB* complementing plasmid pNBGD1003. pNBGD1003 is a shuttle vector derived from pSW4. The locations of the Gram positive and Gram negative replicons (from pUB110 and ColE1, respectively), antibiotic resistance genes *aadD* and *bla,* origin of conjugational transfer *oriT*, constitutively expressed *B. anthracis pagA* promoter (labeled PA promoter) and the *csaB* gene downstream of the promoter are indicated.

We took two parallel approaches to test whether complementation of Δ*csaB* mutant with a wild type copy of *csaB* restores phage sensitivity. First, we introduced a copy of *csaB* in its native locus in the *csaB* deletion strain. In this case, however, we engineered the gene to carry a silent mutation (A693C) creating an *Xma*I site in order to distinguish this strain from the phage-sensitive parent strain. The resulting *csaB-Xma*I mutant strain was verified by PCR to carry the *Xma*I mutation (Figure 5a) and was found to be non-mucoid and phage sensitive. Thus, restoring the wild type allele in its native locus restored the phage sensitive phenotype. Second, complementation was done *in trans* by cloning the wild type *csaB* on a multicopy plasmid (Figure 5b) and transferring the recombinant plasmid (pNBGD1003) into the Δ*csaB* strain via conjugation. In this plasmid, *B. anthracis pagA* promoter was used to drive the constitutive expression of the cloned *csaB*. The resulting recombinant strain was found to be non-mucoid and phage sensitive (Figure 3).

### Transmission electron microscopic analyses of *csaB* mutants and complemented strains

**Figure 6:**
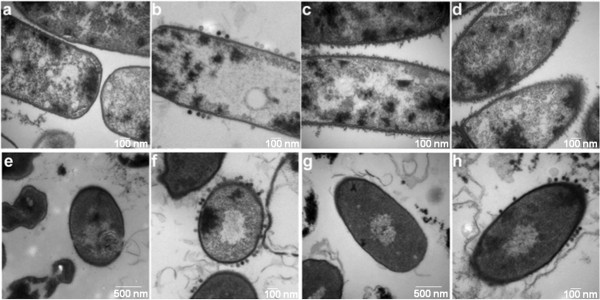
**Transmission electron microscopic images of wt and ∆csaB mutants and the complemented strains.a**) BA663 (csaB+); **b**) BA663+AP50c; **c**) BAP350 (∆csaB); **d**) BAP350 (∆csaB)+AP50c; **e**) BAP533 (csaB+XmaI); **f**) BAP533+AP50c; **g**) BAP350 (∆csaB)+ pBGBN 1003 (csaB+); **h**) BAP350 (∆csaB)+ pBGBN 1003 (csaB+)+AP50c. The scale bar is in the bottom right corner of each figure.

In an earlier study we found that the spontaneous AP50^R^ mutants produced an extracellular matrix and that AP50c phage failed to attach to the mutant bacterial surface [23]. In the current study, we confirmed these observations by transmission electron microscopy (Figure 6a-d). As evident from these images, AP50c phage particles do not attach to the surface of the Δ*csaB* mutant and consequently fail to infect. Phage adsorption and infectivity were regained when Δ*csaB* was complemented *in cis* or *in trans* with the wild type copy of *csaB* (Figure 6e-h). The extracellular secretion is seen only in the mutants and is absent in the wild type and the complemented mutants. The TEM images also revealed that there may be a defective or improperly assembled S-layer, or the S-layer may have been masked by the presence of the extracellular matrix. Further confirmation of the adsorption defect and reversion of this defect in complemented strains was obtained in phage adsorption assays. In the *csaB* mutants (BAP350, BAP356, BAP366, BAP411, BAP435, BAP503) ~62-100% of the phage particles were left in the supernatant whereas in wild type (BA663), Δ*csaB* complemented strains (BAP533 and BGBN001) and the BAS3946 mutants (BAP553, BAP560) most of the phage (>99%) particles were adsorbed and very few particles (~0.1-0.65%) were left in the supernatant. Thus, the defect in phage adsorption was rectified in the complemented strains.

### *csaB* mutants are sensitive to γ phage

Earlier studies have shown that the cell surface protein GamR, which carries a LPXTG motif, is the cellular receptor of the γ phage [12]. We asked whether the cell surface-anchoring defect associated with *csaB* mutants affects GamR in any way, thereby preventing γ adsorption and subsequent infection. Consistent with earlier findings [30], all the mutants and the complemented strains allow infection of γ phage, indicating that mutations in *csaB* do not affect *gamR* expression or bacterial surface localization of GamR protein (Figure 3). Thus, *csaB*-mediated resistance is specific to AP50 phage and is not a general phage resistance mechanism.

## Conclusions

Phage resistance mechanisms are an integral part of the evolutionary arms race between phage and bacteria that has been waged for millions of years. Generally two types of bacterial defense mechanisms are recognized: active (restriction/modification systems, CRISPR/*cas*, and abortive infections) and passive host adaptations [2]. In the latter category, at least two, if not three, types of host adaptations interfering with the phage life cycle can be hypothesized: 1) phage entry, 2) phage growth after entry, and 3) potentially lysogeny can be considered to be the first line of defense against phage infection since lysogens acquire resistance rather quickly and at very high percentage upon infection with a turbid plaque AP50t phage [23]. Mutants of phage that make clear plaques arise in the phage population; these mutants were found to carry two mutations within the phage genome—one in a promoter region and another in a gene of unknown function [23]. Although the clear plaque mutants do not seem to regain the infectivity of a lysogen in the case of AP50 (Sozhamannan, unpublished data), spontaneous resistant strains to AP50c phage arise at very low frequencies in bacterial populations. In mutants that are bacterial surface modified, phage resistance could be caused by masking of the bacterial receptor or by bacterial receptor mutations. Other mutations might affect subsequent steps after the phage DNA enters the cell such as DNA replication, particle assembly, or even lysis of the host. In an effort to understand the passive defense mechanisms, we isolated spontaneous phage resistant bacterial mutants. These mutants secreted an extracellular matrix that appeared to protect the bacterium from phage infection. We hypothesized that this extracellular layer might mask the actual cellular receptor, thereby preventing phage adsorption, since it is a gain of new function due to a mutation and is absent in the wild type strain.

Genetic mapping of a mutation entails linking the phenotype to a genetic locus. Conventional genetic methods use phage-mediated transduction, transformation or conjugation, by which the mutated gene is co-transferred with a known marker and mapped with reference to the known marker. Recombinant DNA and cloning techniques have been used to isolate and identify the gene responsible for a given phenotype. These conventional techniques are time-consuming and are not feasible in many organisms due to the lack of well-defined genetic systems. Recent developments in second-generation sequencing technologies have allowed us to obtain whole genome sequences of mutants and compare the sequences to that of the wild type strain to determine the causal variations. By taking a whole genome sequencing approach, we have traced the causal variation of the phage resistant phenotype to *csaB*. There are two caveats to this approach. First, the sequencing technology used here (Roche/454) is highly error-prone, resulting in approximately 50–100 false-positive variant calls per bacterial genome sequence. Empirically, we have determined that by applying a filter based on the coverage and percentage of variant reads in a given position, positions with 75% or above tend to be true positive variations. We have used a second filter in this study: a biological replicate strategy for ascertaining causal variations. A second caveat to this approach is that we assumed that the phenotype is due to a monogenic trait and that there is a single pathway to give rise to that phenotype. However, despite these limitations, in the current study, we were able to correctly associate the causal variation of the phage resistance phenotype to *csaB* and to further validate this prediction by reconstructing various *csaB* mutants and showing the linkage between phage resistance and *csaB* mutations.

Mesnage et al. have characterized the *csaAB* operon of *B. anthracis* extensively [28]. These authors have proposed a role for *csaB* in cell surface anchoring of S-layer homology (SLH)-containing proteins via pyruvylation of cell wall polysaccharides. Consequently, mutants lacking *csaB* appear to be defective in cell surface anchoring of various SLH proteins including EA1 and Sap. In support of this idea, in *csaB* mutants, Sap and EA1 are conspicuously absent from the cellular fraction and are primarily found in the secretome. These authors also observed additional aberrant cellular morphologies that were replicated in this study, including the accumulation of cell wall material on the cell surface. These authors have attributed this phenotype to the lack of autolysis function in *csaB* mutants [28]. Recently, the role of yet another gene, *tagO,* has been implicated in S-layer protein assembly via binding to secondary cell wall polysaccharide [31]. Given the known function of CsaB in the pyruvylation pathway of polysaccharides, its most probable role in AP50 life cycle may be indirect: in the proper cell surface anchoring of phage receptor protein. The most probable candidates for the bacterial receptor may be Sap and EA1.It is possible that Sap or EA1 is the AP50 receptor, and *csaB* mutants fail to support AP50 adsorption because of the defect in proper cell surface anchoring of the S-layer. The transmission electron micrographs support the idea that the *csaB* mutants lack a well-organized S-layer.

The spontaneous phage resistant mutants isolated in this study carry different types of mutations: SNPs and small insertions leading to nonsense codons, frame-shifts and non-synonymous changes. While a vast majority of these mutations cause CsaB truncation, some other mutants carry non-synonymous changes in the full-length protein. The essentiality of these amino acid residues in the overall structure and function of CsaB is not readily apparent since these mutations fall in regions of very low conservation in the protein alignment (Figure 4 and Additional file 1: Figure S1), but it is possible that the individual amino acid changes may affect protein folding and therefore function. The AP50^R^ mutants derived from 34F_2_ also carried a mutation in BAS3946 besides *csaB*. We have clearly established that the mutation in BAS3946 does not contribute to phage resistance in any way, and it is probably a ‘hitch-hiker’ mutation that was carried in the immediate parent prior to the selection for phage resistance. This result also highlights the power of the next generation sequencers for unbiased mapping of all variations arising in a given bacterial strain under selection pressure or mutagenesis, unlike conventional genetic techniques, which scan a focused area of the genome for mutations.

Another intriguing question that remains is why spontaneous AP50 resistant mutants were not found in the actual phage receptor gene itself, assuming Sap or EA1 is the AP50 phage receptor. It is possible that there are differences in the mutability or essentiality of the genes, although we have already shown that *sap* is not an essential gene for *B. anthracis* viability (Plaut et al., unpublished data). Further characterization of additional genes involved in AP50c life cycle identified by this study is underway. The fact that γ phage and AP50 use different receptors is useful in phage therapy experiments, since development of resistance against both phages would be kept under check when used in combination.

## Methods

### Bacterial strains, plasmids and oligonucleotide primers

Strains and plasmids used in this study are listed in Table 4. The three Sterne strains used in this study were 34F_2_ (obtained from Philip C. Hanna), 7702 (obtained from Theresa M. Koehler) and JB220 (obtained from Richard Calendar). Strain 7702 is designated as BA663 in this study. Derivatives of 34F_2_ that were spontaneously resistant to AP50 are designated S-R1 through S-R9, and those of JB220 are designated J-R1 through J-R8. When necessary, strains were grown in the presence of spectinomycin (100 μg/ml for *E. coli*; 250 μg/ml for *B. anthracis*) or kanamycin (20 μg/ml for *E. coli*; 25 μg/ml for *B. anthracis*). The primers used in various plasmid constructions and Sanger sequencing of PCR amplicons are listed in Additional file 2: Table S1.

### Genome sequencing and variation detection

Whole-genome sequencing (WGS) was performed in the Genome Sequencer FLX (454 Life Sciences/Roche) using FLX or FLX Titanium reagents according to the manufacturer's protocols and instructions [32]. Signal processing of FLX data was performed on the sequencer itself, whereas signal processing of Titanium data was performed off-rig on a Linux cluster of 10 nodes connected via gigabit ethernet. Each node contained eight 64-bit processing cores running at 2.3 GHz with 8 GB of RAM. *De novo* or reference-guided assembly of either FLX or Titanium sequences, preliminary annotation of the genome using the DIYA pipeline [33] and identification of variations were performed using the above-described computer cluster and Sterne (NC 005945) as reference genome as described [27]. A variant flagged by GS Reference Mapper was defined as high confidence if it met the following criteria: a) at least three non-duplicated reads showed the same variation; b) at least one read in each of the forward and reverse orientations showed this variation, unless there are at least five reads with quality scores over 20 (or 30 if the difference involves a 5-mer or higher). A more detailed description of differences is available in the Genome Sequencer Data Analysis Software manual. In this study, a high quality variation is defined as a high confidence (as defined by 454/Roche) and high concordance (≥75% reads have the variation) variation.

### Culture methods and phage assays

All bacterial strains were grown in Luria Broth or Brain Heart Infusion medium, and the phage assays were performed according to published protocols. Phage spot tests were performed as follows: first, triplicate spots of 50 μl of an overnight culture were made on large phage assay agar plates and allowed to dry. Five μl of phages (~10^8^ PFU) AP50c and γ was spotted on two bacterial spots while the third spot received 5 μl of medium. The spots were dried at room temperature and the plates were subsequently incubated at 37°C overnight. Phage adsorption assay was performed as follows: One ml of log phase wild type and mutant *B. anthracis* cells was spun and the pellet was resuspended in 100 μl of AP50c phage and incubated for 30 minutes at room temperature. After addition of 900 μl of phage assay broth, the bacteria-phage complex was filtered through a 0.45 μm syringe filter. The plaque forming units (PFU) in the filtrate was titred using 7702 as the indicator bacterium. The percentage adsorption was calculated against the same volume of unadsorbed phage filtered through the syringe filter.

### Construction of mutants

Two plasmids were engineered to facilitate allelic exchange in *B. anthracis*, as improvements to a published system [29]. Details of the improvements will be published elsewhere. Briefly, pRP1028 and pSS4332 serve the functions of pBKJ236 and pBKJ223, respectively, and contain many of the same features. In pRP1028, the erythromycin resistance of pBKJ236 was replaced with spectinomycin resistance, and in pSS4332, the tetracycline resistance of pBKJ223 was replaced with kanamycin resistance. Allelic exchange constructs derived from pRP1028 were transferred to *B. anthracis*, and plasmid integrants were isolated following a temperature shift [29]. Introduction of pSS4332 into the integrant led to I-*Sce*I-mediated cleavage of the integrated plasmid, stimulating the second crossover event. The presence of the desired changes was confirmed by PCR and sequencing.

Specifically, to reconstruct the point mutations found in the spontaneous AP50^R^ mutants, the mutations and approximately 500 bp of flanking homology on each side were PCR-amplified from the *B. anthracis* strains and cloned into pRP1028. To delete *csaB*, standard cloning methods were used to engineer a construct with the sequence GTG CGG CCCGGG GGA TCT TAA, representing START+one codon+*Xma*I site+two codons+STOP, along with approximately 500 bp of flanking homology.

To restore the wild-type allele to the *csaB* deletion strain, a derivative of pRP1028 was engineered with the *csaB* sequence with the adenosine at position 693 changed to cytosine (creating the *Xma*I site CCCGGG without changing the amino acid sequence), along with approximately 1kb of flanking homology. The change was confirmed by PCR and sequencing, as well as by digestion of the PCR product with *Xma*I (Figure 5a).

To identify conserved residues of CsaB, the nucleotide sequence (BAS0840) was aligned with the 163 proteins in the seed alignment of Pfam family PF04230 (polysaccharide pyruvyl transferase) [34] using ClustalW [35]. The resulting alignment was processed with WebLogo [36] to calculate the consensus sequence and percent conservation at each position (Figure 4 and Additional file 1: Figure S1). The histidine at position 270 of BAS0840 was found to be 100% conserved in these proteins. Standard cloning techniques were used to engineer a derivative of pRP1028 in which the histidine codon (CAT) was changed to alanine (GCT) (Figure 2), flanked by approximately 850 bp of homology. The presence of the H270A change was confirmed by PCR and sequencing.

DNA sequences used to generate ΔBAS3946 and BAS3946 G1024A were synthesized (Gene Oracle, Mountain View, CA). For ΔBAS3946, the sequence included the first 6 codons of the gene, followed by the last 5 codons of the gene and the STOP codon, all flanked by approximately 500 bp of homology. For BAS3946 G1024A, the sequence included the mutation flanked by 500 bp of homology. In each case, DNA was cloned into pRP1028, allelic exchange was performed as described above, and the presence of the desired changes was confirmed by PCR and sequencing.

### Construction of pNBGD1003 and complementation of ΔcsaB

The starting plasmid for pNBGD1003 was pSW4 [37] which was modified by inverse PCR to include a *Not*I-*Nde*I site on either side of the *pagA* promoter using primers 1004 and 1005. The *pagA* promoter was amplified using primers 1006 and 1026, and fragments were digested with *Not*I and *Nde*I and cloned, giving rise to pNBGD1001. Plasmid pNBGD1002 was derived from pNBGD1001 by introducing an origin of transfer, *oriT*, as a *Not*I fragment at the *Not*I site. The *oriT* fragment was PCR amplified from pIN297, which is a derivative of pPL2 [38] using primers 1064 and 1065. Plasmid pNBGD1003 was derived from pNBGD1002 by cloning a *Pml*I- *Bgl*II fragment carrying the *csaB* gene sequences into *Nde*I digested, blunted and *Bam*HI digested pNBGD1002. Plasmid pNBGD1003 was electroporated into SM10 and subsequently transferred into *B. anthracis* Δ*csaB* mutant by conjugation, selecting for polymyxinB and kanamycin resistance as described previously [29]. The resulting ex-conjugants were purified and checked for phage adsorption and infection.

### PCR and Sanger verification of the csaB and BAS3946 genes from AP50^R^ mutants

The DNA sequences spanning the *csaB* (1385 bp) and BAS3946 (1445 bp) genes were PCR amplified from wild type and various mutants (both 34F_2_ and JB220 derivatives), and the PCR amplicons were purified and sequenced on both strands using primers indicated in Table S-1 (BGBN14 through BGBN21). The resulting nucleotide sequences were aligned to the wild type sequences of these two fragments to determine the mutational changes.

### Transmission electron microscopy

Bacterial pellets were fixed in 2.0% glutaraldehyde in 0.1 M sodium cacodylate pH 7.4 overnight at 4°C. Following buffer rinse, samples were post-fixed with 1% osmium tetroxide in 0.1 M sodium cacodylate for 1 hr on ice in the dark. After a brief distilled H_2_O rinse samples were placed in 2% aqueous uranyl acetate (0.22 um filtered) for 1 hour at room temperature in the dark. Following en-bloc staining, cells were dehydrated through a graded series of ethanol to 100% ethanol, transferred through propylene oxide, embedded in Spurr’s low viscosity resin (Polysciences), and cured at 60°C for two days. Sections were cut on a Riechert Ultracut E with a Diatome Diamond knife. Eighty nm sections were picked up on formvar coated 1 × 2 mm copper slot grids and stained with uranyl acetate followed by lead citrate. Grids were viewed on a Hitachi 7600 TEM operating at 80 kV and digital images captured with an AMT 1 K × 1 K CCD camera.

## Competing interests

The authors declare no competing interests.

## Authors’ contributions

KABL, KMW, AB, SD, TL, and MG carried out the whole genome sequencing. KABL, PEC, AA, TDR, and SSo conducted nextgen and Sanger sequence analysis. RDP and SSt carried out deletion and complementation studies. CC performed primer design and microbiological work. AM, VM, and RC participated in the study’s design and coordination and helped draft the manuscript. SSo conceived of the study, participated in its design and coordination and helped draft the manuscript. All authors read and approved the final manuscript.

## Supplementary Material

Additional file 1 **Figure S1.** Sequence alignment of *csaB* and orthologs. BAS0840 was aligned with the Pfam PF04230 seed alignment and percent conservation was determined as described in Methods. Click here for file

Additional file 2 **Table S1.** List of primers used in this study. ^a^ Restriction sites are underlined. Click here for file

## References

[B1] Labrie SJ Samson JE Moineau S Bacteriophage resistance mechanisms Nat Rev Microbiol 2010 8 5 317 327 10.1038/nrmicro2315 20348932

[B2] Stern A Sorek R The phage-host arms race: Shaping the evolution of microbes Bioessays 2011 33 1 43 51 10.1002/bies.201000071 20979102 PMC3274958

[B3] Hagens S Loessner MJ Application of bacteriophages for detection and control of foodborne pathogens Appl Microbiol Biotechnol 2007 76 3 513 519 10.1007/s00253-007-1031-8 17554535

[B4] McAuliffe ORR Fitzgerald GF The new phage biology: from genomics to applications 2007 Norfolk, UK: Caister Academic Press

[B5] McKinstry MER Phages: their role in bacterial pathogenesis and biotechnology Use of phages in therapy and bacterial detection 2005 Washington DC: ASM press

[B6] Petty NK Evans TJ Fineran PC Salmond GP Biotechnological exploitation of bacteriophage research Trends Biotechnol 2007 25 1 7 15 10.1016/j.tibtech.2006.11.003 17113664

[B7] Sulakvelidze A Alavidze Z Morris JG Jr Bacteriophage therapy Antimicrob Agents Chemother 2001 45 3 649 659 10.1128/AAC.45.3.649-659.2001 11181338 PMC90351

[B8] Schuch R Nelson D Fischetti VA A bacteriolytic agent that detects and kills Bacillus anthracis Nature 2002 418 6900 884 889 10.1038/nature01026 12192412

[B9] CDC Center for disease control and prevention: Anthrax Q & A: Diagnosis 2002 http://www.bt.cdc.gov/agent/anthrax/faq/diagnosis.asp

[B10] Abshire TG Brown JE Ezzell JW Production and validation of the use of gamma phage for identification of Bacillus anthracis J Clin Microbiol 2005 43 9 4780 4788 10.1128/JCM.43.9.4780-4788.2005 16145141 PMC1234045

[B11] Brown ER Cherry WB Specific identification of Bacillus anthracis by means of a variant bacteriophage J Infect Dis 1955 96 1 34 39 10.1093/infdis/96.1.34 14354236

[B12] Davison S Couture-Tosi E Candela T Mock M Fouet A Identification of the Bacillus anthracis (gamma) phage receptor J Bacteriol 2005 187 19 6742 6749 10.1128/JB.187.19.6742-6749.2005 16166537 PMC1251577

[B13] Green BD Battisti L Koehler TM Thorne CB Ivins BE Demonstration of a capsule plasmid in Bacillus anthracis Infect Immun 1985 49 2 291 297 3926644 10.1128/iai.49.2.291-297.1985 PMC262013

[B14] Ruhfel RE Robillard NJ Thorne CB Interspecies transduction of plasmids among Bacillus anthracis, B. cereus, and B. thuringiensis J Bacteriol 1984 157 3 708 711 6421798 10.1128/jb.157.3.708-711.1984 PMC215315

[B15] Thorne CB Transduction in Bacillus cereus and Bacillus anthracis Bacteriol Rev 1968 32 4 Pt 1 358 361 4974088 PMC408306

[B16] Yelton DB Thorne CB Transduction in Bacillus cereus by each of two bacteriophages J Bacteriol 1970 102 2 573 579 4986764 10.1128/jb.102.2.573-579.1970 PMC247587

[B17] Walter TM Aronson AI Transduction of certain genes by an autonomously replicating Bacillus thuringiensis phage Appl Environ Microbiol 1991 57 4 1000 1005 2059027 10.1128/aem.57.4.1000-1005.1991 PMC182836

[B18] Nagy E A highly specific phage attacking Bacillus anthracis strain Sterne Acta Microbiol Acad Sci Hung 1974 21 3–4 257 263 4215291

[B19] Nagy E Pragai B Ivanovics G Characteristics of phage AP50, an RNA phage containing phospholipids J Gen Virol 1976 32 1 129 132 10.1099/0022-1317-32-1-129 822131

[B20] Nagy E Ivanovics G Association of probable defective phage particles with lysis by bacteriophage AP50 in Bacillus anthracis J Gen Microbiol 1977 102 1 215 219 10.1099/00221287-102-1-215 410906

[B21] Nagy E Herczegh O Ivanova N Lipid-containing anthrax phage AP50: Structural proteins and life cycle J Gen Virol 1982 62 323 329 10.1099/0022-1317-62-2-323

[B22] Nagy E Ivanovics G Anthrax-specific "AP 50-like" phages isolated from Bacillus cereus strains Acta Microbiol Acad Sci Hung 1982 29 2 89 98 6814198

[B23] Sozhamannan S McKinstry M Lentz SM Jalasvuori M McAfee F Smith A Dabbs J Ackermann HW Bamford JK Mateczun A Molecular characterization of a variant of Bacillus anthracis-specific phage AP50 with improved bacteriolytic activity Appl Environ Microbiol 2008 74 21 6792 6796 10.1128/AEM.01124-08 18791014 PMC2576697

[B24] Davis BM Waldor MK High-throughput sequencing reveals suppressors of Vibrio cholerae rpoE mutations: one fewer porin is enough Nucleic Acids Res 2009 37 17 5757 5767 10.1093/nar/gkp568 19620211 PMC2761261

[B25] Hobert O The impact of whole genome sequencing on model system genetics: get ready for the ride Genetics 2010 184 2 317 319 10.1534/genetics.109.112938 20103786 PMC2828713

[B26] Feng J Lupien A Gingras H Wasserscheid J Dewar K Legare D Ouellette M Genome sequencing of linezolid-resistant Streptococcus pneumoniae mutants reveals novel mechanisms of resistance Genome Res 2009 19 7 1214 1223 10.1101/gr.089342.108 19351617 PMC2704432

[B27] Chen PE Willner KM Butani A Dorsey S George M Stewart A Lentz SM Cook CE Akmal A Price LB Rapid identification of genetic modifications in Bacillus anthracis using whole genome draft sequences generated by 454 pyrosequencing PLoS One 2010 5 8 10.1371/journal.pone.0012397 PMC2928293 20811637

[B28] Mesnage S Fontaine T Mignot T Delepierre M Mock M Fouet A Bacterial SLH domain proteins are non-covalently anchored to the cell surface via a conserved mechanism involving wall polysaccharide pyruvylation EMBO J 2000 19 17 4473 4484 10.1093/emboj/19.17.4473 10970841 PMC302060

[B29] Janes BK Stibitz S Routine markerless gene replacement in Bacillus anthracis Infect Immun 2006 74 3 1949 1953 10.1128/IAI.74.3.1949-1953.2006 16495572 PMC1418658

[B30] Fouet A The surface of Bacillus anthracis Mol Aspects Med 2009 30 6 374 385 10.1016/j.mam.2009.07.001 19607856

[B31] Kern J Ryan C Faull K Schneewind O Bacillus anthracis surface-layer proteins assemble by binding to the secondary cell wall polysaccharide in a manner that requires csaB and tagO J Mol Biol 2010 401 5 757 775 10.1016/j.jmb.2010.06.059 20603129 PMC4652593

[B32] Margulies M Egholm M Altman WE Attiya S Bader JS Bemben LA Berka J Braverman MS Chen YJ Chen Z Genome sequencing in microfabricated high-density picolitre reactors Nature 2005 437 7057 376 380 16056220 10.1038/nature03959 PMC1464427

[B33] Stewart AC Osborne B Read TD DIYA: a bacterial annotation pipeline for any genomics lab Bioinformatics 2009 25 7 962 963 10.1093/bioinformatics/btp097 19254921 PMC2660880

[B34] Finn RD Mistry J Tate J Coggill P Heger A Pollington JE Gavin OL Gunasekaran P Ceric G Forslund K The Pfam protein families database Nucleic Acids Res 2010 38 (Database issue) D211 222 19920124 10.1093/nar/gkp985 PMC2808889

[B35] Thompson JD Higgins DG Gibson TJ CLUSTAL W: improving the sensitivity of progressive multiple sequence alignment through sequence weighting, position-specific gap penalties and weight matrix choice Nucleic Acids Res 1994 22 22 4673 4680 10.1093/nar/22.22.4673 7984417 PMC308517

[B36] Crooks GE Hon G Chandonia JM Brenner SE WebLogo: a sequence logo generator Genome Res 2004 14 6 1188 1190 10.1101/gr.849004 15173120 PMC419797

[B37] Pomerantsev AP Kalnin KV Osorio M Leppla SH Phosphatidylcholine-specific phospholipase C and sphingomyelinase activities in bacteria of the Bacillus cereus group Infect Immun 2003 71 11 6591 6606 10.1128/IAI.71.11.6591-6606.2003 14573681 PMC219565

[B38] Lauer P Chow MY Loessner MJ Portnoy DA Calendar R Construction, characterization, and use of two Listeria monocytogenes site-specific phage integration vectors J Bacteriol 2002 184 15 4177 4186 10.1128/JB.184.15.4177-4186.2002 12107135 PMC135211

[B39] Simon R Priefer UB Puhler A A Broad Host Range Mobilization System for In Vivo Genetic Engineering: Transposon Mutagenesis in Gram Negative Bacteria Nat Biotech 1983 1 9 784 791 10.1038/nbt1183-784

