# Supplementary figures and images for: Whole genome sequencing of phage resistant Bacillus anthracis mutants reveals an essential role for cell surface anchoring protein CsaB in phage AP50c adsorption
Source: Virol J. 2012 Oct 26;9:246. doi: 10.1186/1743-422X-9-246 (PMC3545897; doi:10.1186/1743-422X-9-246)

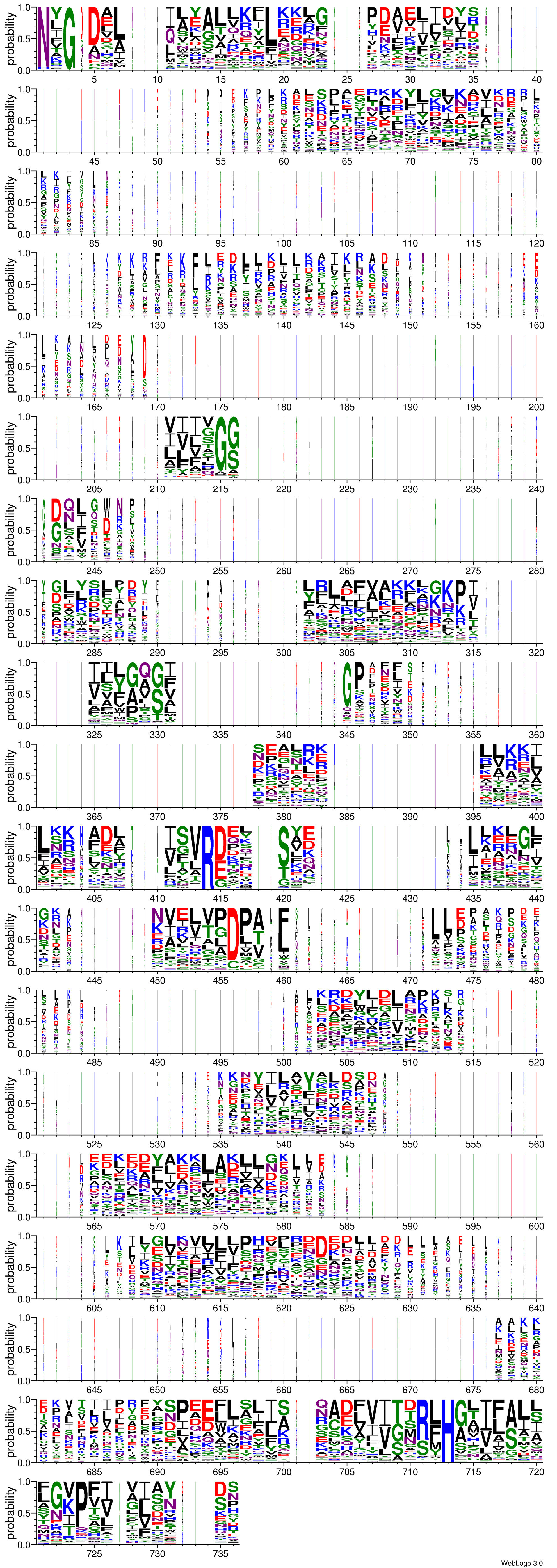

Supplement: Additional file 1 — Figure S1. Sequence alignment of csaB and orthologs. BAS0840 was aligned with the Pfam PF04230 seed alignment and percent conservation was determined as described in Methods. [file 1743-422X-9-246-S1.jpeg]
