# Supplementary material for: Whole genome sequencing of phage resistant Bacillus anthracis mutants reveals an essential role for cell surface anchoring protein CsaB in phage AP50c adsorption
Source: Virol J. 2012 Oct 26;9:246. doi: 10.1186/1743-422X-9-246 (PMC3545897; doi:10.1186/1743-422X-9-246)
Supplement: Additional file 2 — Table S1. List of primers used in this study. a Restriction sites are underlined. [file 1743-422X-9-246-S2.doc]

**Table S-1: List of primers used in this study**

| Primer | Sequence (5' to 3') | Purpose/Application(s) | Restriction site(s)^a^ |
| --- | --- | --- | --- |
| 231 | TATGGTCTC GGATCCAGGAATTAACCGTTCTAAGCAAGCG | Δ*csaB*; A693C | *Bsa*I, *Bam*HI |
| 232 | TATGGTCTC CCCGGGCCGCACTCTTAATCTCCTCCAAC | Δ*csaB* | *Bsa*I, *Xma*I |
| 233 | TATGGTCTC CCCGGGGGATCTTAATTTAAGAGGACATCCTC | Δ*csaB* | *Bsa*I, *Xma*I |
| 234 | TATGGTCTC CGGCCGAGAATCATAAAGAAATCATACACCC | Δ*csaB*; H270A; A693C | *Bsa*I, *Eag*I |
| 236 | TATGGTCTC CGGCCGCCTCTTAAATTAAGATCCCATTCCTC | amplify S-R1, S-R3, S-R4, and S-R6 | *Bsa*I, *Eag*I |
| 237 | TATGGTCTC GGATCCCGATAACAATCTGGCTCATGGCAC | diagnose Δ*csaB* and A693C; amplify S-R3, S-R4, and S-R6 | *Bsa*I, *Bam*HI |
| 238 | TATGGTCTC GGATCCTTGCCTTGGGGTAATTACGTCACTG | amplify S-R1 | *Bsa*I, *Bam*HI |
| 251 | CTGCTGCTACTGGAGAAACAACACC | diagnose Δ*csaB* deletion and A693C | none |
| 301 | TATGGTCTC GGATCCTGAGTCCATAAATGAGTATGGC | H270A | *Bsa*I, *Bam*HI |
| 304 | TATGGTCTCTGCAAGTCTCATACCGATTAGAAGCG | H270A | *Bsa*I |
| 305 | TATGGTCTCTTTGCTGCGCTCATACTATCTGCCGTTGC | H270A | *Bsa*I |
| 306 | TATGGTCTCACCGGGATGCATTTTGATCAAATGGTCC | A693C | *Bsa*I |
| 307 | TATGGTCTCACCGGGATATTATTAATTTAATGGGAG | A693C | *Bsa*I |
| 402 | TTACAAGTGCATGATCGTCC | diagnose BAS3946 G1024A | none |
| 403 | AAGGATGAGGCCAATTCACC | diagnose BAS3946 G1024A | none |
| 404 | CGGTAGGGAATGAAAGTGTG | diagnose ΔBAS3946 | none |
| 405 | CTTAAGTCATACCATTGACG | diagnose ΔBAS3946 | none |
| 1004 | GGGCCCCATATGGGATCCCGGGGGGTAAG | pSW-PA promoter_forward | *Nde*I |
| 1005 | GGGCCCGCGGCCGCTTATGTTTAATAGGATGAATCCGAA CCTCATTACAC | pSW-PA_promoter_reverse | *Not*I |
| 1006 | CCCGGGGCGGCCGCTAAATTCTTTTTTATGTTATATATT TATAAAAG | prPA-forward | *Not*I |
| 1026 | CCCGGGCATATGCGTTCTCCTTTTTGTATAAAATTAAAT TTATA | prPA_reverse_NdeI | *Nde*I |
| 1064 | GGGCCCGCGGCCGCCGCTTGCCCTCATCTGTTAC | *oriT*_*Not*I_for | *Not*I |
| 1065 | GGGCCCGCGGCCGCCTCTCGCCTGTCCCCTCAGT | *oriT*_*Not*T_rev | *Not*I |
| BGBN10 | GGGCCCCACGTGCGGTTAGTTTTATCAGGATATTATGG | *csaB* forward | *Pml*I |
| BGBN11 | GGGCCCAGATCTTTAAGATCCCATTCCTCTTTTTTTGAA CTCTTTTG | *csaB* reverse | *Bgl*II |
| BGBN14 | ATGAGTCCATAAATGAGTATGGCTATTTATATG | *csaB*_upstream |  |
| BGBN15 | ATACATCTTATCTCTCAAACTTCTTTTTTATGAG | *csaB* downstream |  |
| BGBN16 | AATATGGTATCCATCACGCTTTAATTGTTTCAG | *csaB-*sequencing_primer-rev |  |
| BGBN17 | GGTATTAAGAAGGATATTGAACTAGTTCCAGATC | *csaB_*sequencing_primer_for |  |
| BGBN18 | TGCTAATTCTCGCAGCATTACGCTGACAG | Enolase forward |  |
| BGBN19 | TTTACTTCTTCAGCTTCTGGTGGTGCGAATTTC | Enolase reverse |  |
| BGBN20 | TGCATTAACACCACTTACGAACCGCATTGTATC | Enolase_sequencing_primer_up |  |
| BGBN21 | CTCCAGCTTGCACAGCACGTTTTGCATTTTC | Enolase_sqeuencing_primer_dn |  |

^a^ Restriction sites are underlined.
